# (2S*,5R*)-2,5-Dimethyl-1,4-bis­(pyridin-2-ylmeth­yl)piperazine

**DOI:** 10.1107/S160053681301605X

**Published:** 2013-06-15

**Authors:** Christopher Goh, Lilliana S. Morris, Michael P. Girouard, Tamuka Chidanguro, Jerry P. Jasinski

**Affiliations:** a Department of Chemistry, Williams College, Williamstown, MA 01267, USA; b Department of Chemistry, Keene State College, 229 Main Street, Keene, NH 03435-2001, USA

## Abstract

The title compound, C_18_H_24_N_4_, resides on a crystallographic inversion centre, so that the asymmetric unit comprises one half-mol­ecule. The piperazine ring adopts a chair conformation, with the mean planes of the two equatorial pyridine rings parallel to each other and separated by 2.54 (3) Å. No classical hydrogen bonds are observed.

## Related literature

For related work on the synthesis of tetra­dentate pyridine-piperazine ligands and for metal complexes of these ligands, see: Geiger *et al.* (2011 ▶); Ostermeier *et al.* (2006 ▶, 2009 ▶); Nam (2007 ▶); Huuskonen *et al.* (1995 ▶); Que & Tolman (2008 ▶); Ratilainen *et al.* (1999 ▶); Fuji *et al.* (1996 ▶). For the synthesis, see: Halfen *et al.* (2000 ▶).
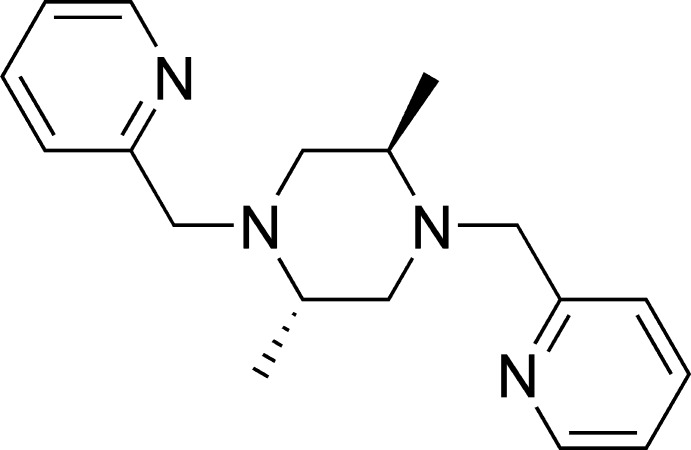


## Experimental

### 

#### Crystal data

C_18_H_24_N_4_
*M* *_r_* = 296.41
Orthorhombic, 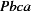

*a* = 9.4097 (5) Å
*b* = 9.2191 (5) Å
*c* = 18.7473 (9) Å
*V* = 1626.29 (14) Å^3^
*Z* = 4
Cu *K*α radiation
μ = 0.57 mm^−1^
*T* = 173 K
0.22 × 0.18 × 0.04 mm

#### Data collection

Agilent Xcalibur (Eos, Gemini) diffractometer
Absorption correction: multi-scan (*CrysAlis PRO* and *CrysAlis RED*; Agilent, 2012 ▶) *T*_min_ = 0.817, *T*_max_ = 1.000
10101 measured reflections
1545 independent reflections
1392 reflections with *I* > 2σ(*I*)
*R*_int_ = 0.064

#### Refinement

*R*[*F* ^2^ > 2σ(*F* ^2^)] = 0.044
*wR*(*F* ^2^) = 0.131
*S* = 1.07
1545 reflections
102 parameters
H-atom parameters constrained
Δρ_max_ = 0.24 e Å^−3^
Δρ_min_ = −0.19 e Å^−3^

### 

Data collection: *CrysAlis PRO* (Agilent, 2012 ▶); cell refinement: *CrysAlis PRO*; data reduction: *CrysAlis RED* (Agilent, 2012 ▶); program(s) used to solve structure: *SHELXS97* (Sheldrick, 2008 ▶); program(s) used to refine structure: *SHELXL2012* (Sheldrick, 2008 ▶); molecular graphics: *OLEX2* (Dolomanov *et al.*, 2009 ▶); software used to prepare material for publication: *OLEX2*.

## Supplementary Material

Crystal structure: contains datablock(s) global, I. DOI: 10.1107/S160053681301605X/fj2633sup1.cif

Structure factors: contains datablock(s) I. DOI: 10.1107/S160053681301605X/fj2633Isup2.hkl

Supplementary material file. DOI: 10.1107/S160053681301605X/fj2633Isup3.cml

Additional supplementary materials:  crystallographic information; 3D view; checkCIF report

## Figures and Tables

**Fig. 1.**
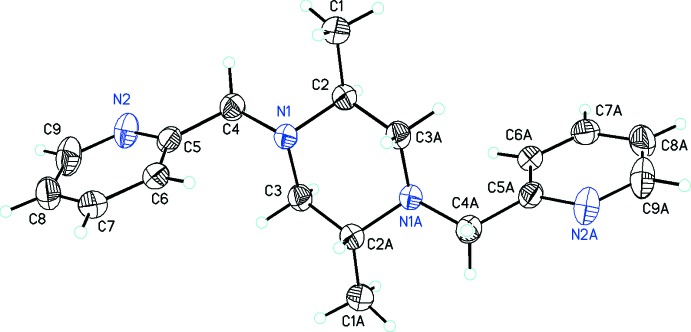
Molecular structure of the title compound showing the atom labeling scheme and 30% probability displacement ellipsoids.

**Fig. 2.**
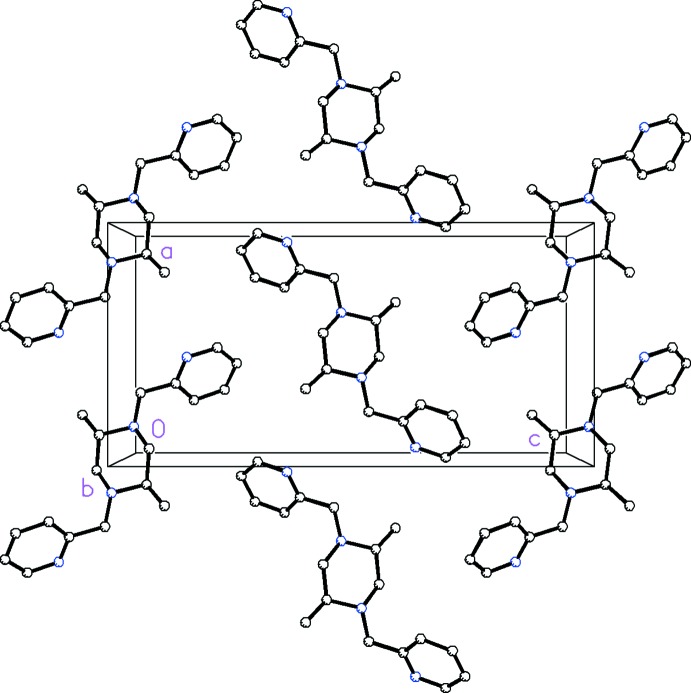
Packing diagram of the title compound viewed along the b axis. H atoms have been removed for clarity.

## supplementary crystallographic information

### Comment

Multidentate ligands containing pyridine and amine donor moieties have applications in metal-catalyzed oxidations and in the design of macrocyclic metal-binding receptors. Examples include the manganese, iron, and copper complexes of tetradentate pyridine and amine ligands for biologically-inspired oxidations (Geiger *et al.*, 2011; Ostermeier *et al.*, 2009; Que *et al.*, 2008; Nam, 2007; Ostermeier *et al.*, 2006), copper complexes of pyridine-diazacycloalkanes as catalysts for the aziridination of alkenes (Halfen *et al.*, 2000) and macrocyclic piperazinacyclophanes as complexation agents for a host of metals (Ratilainen *et al.*, 1999; Fuji *et al.*, 1996; Huuskonen *et al.*, 1995). Our group has been interested in the use of neutral tetradentate hetero-aromatic-amine ligands in metal-catalyzed oxidations. Here we report the synthesis and crystal structure of the meso form of the tetradentate ligand, (I), (2S,5R)-2,5-dimethyl-1,4-bis(pyridin-2-ylmethyl)piperazine (Fig. 1).

In the asymmetric unit of the title compound, C_18_H_24_N_4_, (I), a piperazine ring (N1/C2/C3A/N1A/C2A/C3) is formed by a center of symmetry connecting each half (N/C/C) to a methyl group and pyridine ring at the 2,5 and 1,4 positions, respectively. The piperazine ring adopts a chair conformation with puckering parameters Q = 0.5804 (13)Å, θ = 0.00 (1)°, φ = 0.0000°. The mean planes of the two equatorial pyridine rings are parallel to each other and separated by 2.54 (3)Å, respectively. In the formation of this neutral tetradentate hetero-aromatic-amine ligand no classical hydrogen bonds are observed (Fig. 2).

### Experimental

The title compound was synthesized under a dinitrogen atmosphere by modifications of a previously published protocol (Halfen *et al.*, 2000). 2-picolyl chloride hydrochloride (2.87 g, 17.5 mmol) and triethylamine (4.88 mL, 35.0 mmol) were added to a suspension of (2R, 5S)-2,5-dimethylpiperazine (1.00 g, 8.76 mmol) in 30 mL of acetonitrile to form a slurry. The mixture was allowed to stir for 48 hours at room temperature and then treated with 100 mL of 1 M sodium hydroxide. The product was extracted with three portions of 50 mL of CH_2_Cl_2_. The combined fractions were dried with MgSO_4_, filtered and the solvent removed to yield the crude product as a brown solid. Further purification by column chromatography with a Biotage Isolera^TM^ Flash Purification System using a silica cartridge and a gradient of ethyl acetate and a mixture of ethyl acetate/methanol/triethylamine (90/5/5), followed by solvent removal yielded the pure product as a faintly brown transparent solid (1.40 g, 54% yield). Crystallization by evaporation from a concentrated diethyl ether solution led to isolation of crystals suitable for X-ray analysis (m.p.: 405–406K).

### Refinement

All of the H atoms were placed in their calculated positions and then refined using the riding model with Atom—H lengths of 0.95Å (CH), 0.99Å (CH_2_) or 0.98Å (CH_3_). Isotropic displacement parameters for these atoms were set to 1.2 (CH, CH_2_) or 1.5 (CH_3_times *U*_eq_ of the parent atom. Ternary CH were refined with riding coordinates: C2(H2), secondary CH_2_ refined with riding coordinates: C3(H3A,H3B), C4(H4A,H4B), aromatic/amide H refined with riding coordinates: C6(H6), C7(H7), C8(H8), C9(H9), idealised Me refined as rotating group: C1(H1A,H1B,H1C).

### Crystal data

**Table:**

C_18_H_24_N_4_	*D*_x_ = 1.211 Mg m^−^^3^
*M**_r_* = 296.41	Cu *K*α radiation, λ = 1.5418 Å
Orthorhombic, *P**b**c**a*	Cell parameters from 4359 reflections
*a* = 9.4097 (5) Å	θ = 4.7–70.6°
*b* = 9.2191 (5) Å	µ = 0.57 mm^−^^1^
*c* = 18.7473 (9) Å	*T* = 173 K
*V* = 1626.29 (14) Å^3^	Block, colourless
*Z* = 4	0.22 × 0.18 × 0.04 mm
*F*(000) = 640	

### Data collection

**Table:**

Agilent Xcalibur (Eos, Gemini) diffractometer	1545 independent reflections
Radiation source: Enhance (Cu) X-ray Source	1392 reflections with *I* > 2σ(*I*)
Graphite monochromator	*R*_int_ = 0.064
Detector resolution: 16.0416 pixels mm^-1^	θ_max_ = 70.7°, θ_min_ = 6.7°
ω scans	*h* = −10→11
Absorption correction: multi-scan (*CrysAlis PRO* and *CrysAlis RED*; Agilent, 2012)	*k* = −8→11
*T*_min_ = 0.817, *T*_max_ = 1.000	*l* = −22→21
10101 measured reflections	

### Refinement

**Table:**

Refinement on *F*^2^	Hydrogen site location: inferred from neighbouring sites
Least-squares matrix: full	H-atom parameters constrained
*R*[*F*^2^ > 2σ(*F*^2^)] = 0.044	*w* = 1/[σ^2^(*F*_o_^2^) + (0.0744*P*)^2^ + 0.3744*P*] where *P* = (*F*_o_^2^ + 2*F*_c_^2^)/3
*wR*(*F*^2^) = 0.131	(Δ/σ)_max_ < 0.001
*S* = 1.07	Δρ_max_ = 0.24 e Å^−^^3^
1545 reflections	Δρ_min_ = −0.19 e Å^−^^3^
102 parameters	Extinction correction: *SHELXL2012* (Sheldrick, 2008), Fc^*^=kFc[1+0.001xFc^2^λ^3^/sin(2θ)]^-1/4^
0 restraints	Extinction coefficient: 0.0056 (9)
Primary atom site location: structure-invariant direct methods	

### Special details

**Table:**

Experimental. ^1^H-NMR (CDCl_3_, 298 K): δ 8.55 (d, J = 3.5 Hz, 2H, py), 7.65 (m, 2H, py), 7.44 (d, J = 7.5 Hz, 2H, py), 7.15 (m, 2H, py), 4.15 (d, J = 14 Hz, 2 H, py-CH_2_N), 3.38 (d, J = 14 Hz, 2H, py-CH_2_N), 2.68 (m, 2H, NCH_2_), 2.50 (m, 2H, NCH), 2.14 (m, 2H, NCH_2_), 1.07 (d, J = 6.1 Hz, 6H, CH_3_) ppm. ^13^C-NMR (CDCl_3_, 298K): δ 159.6 (py), 150.0 (py), 136.3 (py), 123.2 (py), 121.8 (py), 60.5, 59.7, 56.0, 17.8 (CH_3_) ppm. MS: m/z 204 (py-CH_2_N_2_C_6_H_12_), m/z 175 (py-CH_2_NC_5_H_9_), m/z 149 (py-CH_2_NC_3_H_7_), m/z 135.0 (py-CH_2_NC_2_H_4_), m/z 112 (N_2_C_6_H_12_), m/z 93 (py-CH_3_).
Geometry. All esds (except the esd in the dihedral angle between two l.s. planes) are estimated using the full covariance matrix. The cell esds are taken into account individually in the estimation of esds in distances, angles and torsion angles; correlations between esds in cell parameters are only used when they are defined by crystal symmetry. An approximate (isotropic) treatment of cell esds is used for estimating esds involving l.s. planes.

### Fractional atomic coordinates and isotropic or equivalent isotropic displacement parameters (Å^2^)

**Table:**

	*x*	*y*	*z*	*U*_iso_*/*U*_eq_	
N1	0.35844 (11)	0.53852 (12)	0.52226 (5)	0.0296 (3)	
N2	0.05342 (13)	0.49301 (14)	0.64052 (7)	0.0433 (4)	
C1	0.31099 (16)	0.64089 (18)	0.40179 (8)	0.0431 (4)	
H1A	0.3227	0.7380	0.4222	0.065*	
H1B	0.2100	0.6148	0.4018	0.065*	
H1C	0.3469	0.6403	0.3527	0.065*	
C2	0.39382 (14)	0.53155 (14)	0.44625 (7)	0.0309 (4)	
H2	0.3740	0.4316	0.4281	0.037*	
C3	0.44872 (13)	0.43666 (15)	0.56137 (7)	0.0316 (4)	
H3A	0.4297	0.3372	0.5439	0.038*	
H3B	0.4230	0.4399	0.6126	0.038*	
C4	0.20889 (13)	0.50891 (17)	0.53835 (7)	0.0352 (4)	
H4A	0.1907	0.4036	0.5333	0.042*	
H4B	0.1482	0.5604	0.5034	0.042*	
C5	0.16893 (14)	0.55639 (15)	0.61287 (7)	0.0319 (3)	
C6	0.24476 (15)	0.66281 (16)	0.64872 (7)	0.0339 (4)	
H6	0.3263	0.7057	0.6275	0.041*	
C7	0.20015 (15)	0.70565 (18)	0.71574 (7)	0.0411 (4)	
H7	0.2508	0.7779	0.7414	0.049*	
C8	0.08112 (17)	0.64193 (19)	0.74468 (8)	0.0476 (4)	
H8	0.0476	0.6693	0.7905	0.057*	
C9	0.01202 (18)	0.53765 (18)	0.70546 (9)	0.0507 (5)	
H9	−0.0704	0.4943	0.7256	0.061*	

### Atomic displacement parameters (Å^2^)

**Table:**

	*U*^11^	*U*^22^	*U*^33^	*U*^12^	*U*^13^	*U*^23^
N1	0.0247 (6)	0.0370 (6)	0.0270 (6)	0.0004 (4)	0.0020 (4)	0.0000 (4)
N2	0.0353 (7)	0.0432 (7)	0.0513 (8)	−0.0014 (5)	0.0150 (5)	−0.0023 (6)
C1	0.0377 (8)	0.0577 (10)	0.0340 (7)	0.0097 (7)	0.0010 (6)	0.0065 (6)
C2	0.0296 (7)	0.0365 (7)	0.0266 (7)	0.0013 (5)	0.0006 (5)	−0.0020 (5)
C3	0.0315 (7)	0.0345 (7)	0.0288 (7)	−0.0004 (5)	0.0043 (5)	0.0021 (5)
C4	0.0268 (7)	0.0452 (8)	0.0335 (7)	−0.0029 (5)	0.0015 (5)	−0.0039 (6)
C5	0.0253 (6)	0.0356 (7)	0.0347 (7)	0.0050 (5)	0.0028 (5)	0.0033 (5)
C6	0.0279 (7)	0.0420 (8)	0.0317 (7)	0.0033 (5)	−0.0001 (5)	0.0019 (5)
C7	0.0380 (8)	0.0503 (9)	0.0349 (7)	0.0098 (6)	−0.0043 (6)	−0.0040 (6)
C8	0.0485 (9)	0.0590 (10)	0.0353 (8)	0.0155 (7)	0.0111 (6)	0.0009 (7)
C9	0.0447 (9)	0.0509 (10)	0.0566 (10)	0.0024 (7)	0.0245 (8)	0.0042 (8)

### Geometric parameters (Å, º)

**Table:**

N1—C2	1.4648 (16)	C3—H3B	0.9900
N1—C3	1.4633 (17)	C4—H4A	0.9900
N1—C4	1.4649 (17)	C4—H4B	0.9900
N2—C5	1.3385 (18)	C4—C5	1.5115 (18)
N2—C9	1.343 (2)	C5—C6	1.387 (2)
C1—H1A	0.9800	C6—H6	0.9500
C1—H1B	0.9800	C6—C7	1.3824 (19)
C1—H1C	0.9800	C7—H7	0.9500
C1—C2	1.5226 (19)	C7—C8	1.376 (2)
C2—H2	1.0000	C8—H8	0.9500
C2—C3^i^	1.5171 (17)	C8—C9	1.374 (3)
C3—C2^i^	1.5171 (17)	C9—H9	0.9500
C3—H3A	0.9900		
			
C2—N1—C4	114.24 (10)	N1—C4—H4A	109.2
C3—N1—C2	109.11 (10)	N1—C4—H4B	109.2
C3—N1—C4	109.57 (10)	N1—C4—C5	112.04 (11)
C5—N2—C9	116.93 (14)	H4A—C4—H4B	107.9
H1A—C1—H1B	109.5	C5—C4—H4A	109.2
H1A—C1—H1C	109.5	C5—C4—H4B	109.2
H1B—C1—H1C	109.5	N2—C5—C4	115.69 (12)
C2—C1—H1A	109.5	N2—C5—C6	122.62 (13)
C2—C1—H1B	109.5	C6—C5—C4	121.65 (12)
C2—C1—H1C	109.5	C5—C6—H6	120.4
N1—C2—C1	112.78 (11)	C7—C6—C5	119.10 (13)
N1—C2—H2	109.2	C7—C6—H6	120.4
N1—C2—C3^i^	107.77 (10)	C6—C7—H7	120.5
C1—C2—H2	109.2	C8—C7—C6	118.91 (14)
C3^i^—C2—C1	108.69 (11)	C8—C7—H7	120.5
C3^i^—C2—H2	109.2	C7—C8—H8	120.9
N1—C3—C2^i^	113.32 (11)	C9—C8—C7	118.22 (14)
N1—C3—H3A	108.9	C9—C8—H8	120.9
N1—C3—H3B	108.9	N2—C9—C8	124.22 (15)
C2^i^—C3—H3A	108.9	N2—C9—H9	117.9
C2^i^—C3—H3B	108.9	C8—C9—H9	117.9
H3A—C3—H3B	107.7		
			
N1—C4—C5—N2	−158.76 (12)	C4—N1—C2—C3^i^	−179.58 (11)
N1—C4—C5—C6	23.55 (18)	C4—N1—C3—C2^i^	−174.32 (11)
N2—C5—C6—C7	0.1 (2)	C4—C5—C6—C7	177.62 (12)
C2—N1—C3—C2^i^	59.93 (15)	C5—N2—C9—C8	−0.6 (3)
C2—N1—C4—C5	−164.47 (11)	C5—C6—C7—C8	−0.4 (2)
C3—N1—C2—C1	−176.54 (12)	C6—C7—C8—C9	0.2 (2)
C3—N1—C2—C3^i^	−56.57 (14)	C7—C8—C9—N2	0.3 (3)
C3—N1—C4—C5	72.78 (14)	C9—N2—C5—C4	−177.26 (13)
C4—N1—C2—C1	60.45 (15)	C9—N2—C5—C6	0.4 (2)

Symmetry code: (i) −*x*+1, −*y*+1, −*z*+1.
